# Demand Potential for Agricultural Trade between China and Countries along the “Belt and Road”

**DOI:** 10.3390/foods13193210

**Published:** 2024-10-09

**Authors:** Lunzheng Zhou, Guangji Tong, Jiaguo Qi, Lu He

**Affiliations:** 1 College of Economics and Management, East China Jiaotong University, Nanchang 330013, China; 3640@ecjtu.edu.cn; 2 College of Economics and Management, Northeast Forestry University, Harbin 150040, China; tonggj63@nefu.edu.cn; 3 Center for Global Change and Earth Observations, Michigan State University, East Lansing, MI 48824, USA; qi@msu.edu; 4 College of Economics and Management, Harbin Institute of Technology, Harbin 150001, China

**Keywords:** “Belt and Road”, food safety, demand potential, demand elasticity

## Abstract

International agricultural markets are an important part of the global food resource chain. Tapping into the potential of agricultural trade between China and countries along the “Belt and Road” (B&R) is conducive to safeguarding China’s and the world’s food security, but there is less literature on the potential of bilateral trade demand. This paper ranks the B&R countries according to the scale of imports and exports, and calculates the elasticity of demand for imports, the elasticity of substitution for exports, and, finally, the potential of elasticity of demand for trade between China and the major B&R countries. The results show that China’s agricultural export potential to major B&R countries is ranked as follows: Indonesia, Thailand, Russia, Poland, Turkey, Vietnam, Malaysia, Ukraine, India, and Singapore. The major B&R countries are also ranked in terms of their export potential to China: Vietnam, India, Ukraine, Russia, Malaysia, Thailand, Indonesia, Poland, Singapore, and Turkey. The findings of this paper provide a decision-making basis for promoting agricultural trade between China and B&R countries.

## 1. Introduction

Food security is the foundation of national security. China is the world’s largest importer of agricultural products and imports equivalent to nearly half of the domestic sowing area. The use of international and domestic markets, using the theory of “two markets, two kinds of resources”, is the only option to protect China’s food security. Internationally, China’s agricultural imports are highly concentrated, with nearly 50 percent coming from the Americas. At the same time, simulated crop yield losses under the most severe climate change scenarios range from 7% to 23% if no adaptative measures are taken [1]. For the Russian–Ukrainian conflict, in the “worst case” scenario simulation, more than 28% of countries will face supply shortages in excess of their domestic reserves, while more than 50% of countries will lose more than 40% of their reserves [2]. In light of these uncertainties and the worsening global food supply crisis, identifying how to enhance and diversify China’s imports of agricultural products and establishing trade initiatives are necessary to solve the current problem.

In 2013, Chinese President Xi Jinping first proposed the construction of the New Silk Road Economic Belt and the 21st Century Maritime Silk Road, also known as the Belt and Road Initiative. The Belt and Road Initiative, which upholds the development concept of a community of human destiny, is currently the largest global public good. The “Belt and Road” initiative was first proposed in China, and the central focus of China’s agricultural import trade has gradually shifted to B&R countries [3]. Especially in the period of trade friction between China and the United States, China has further strengthened agricultural cooperation with B&R countries, reducing the risks associated with soybean imports [4]. In the face of the world’s uncertainties, can China’s trade of agricultural products be further shifted to B&R countries? What is the potential for demand for agricultural products between China and B&R countries, especially in terms of market price movements? Therefore, promoting agricultural trade between China and B&R countries and tapping into the potential of bilateral trade play an important role in guaranteeing food security both globally and in China [5].

## 2. Literature Review and Analysis of Previous Studies

The existing literature on trade potential is based on the index method and the gravity model method, with the index method combining five indicators, namely the comparative advantage index (RCA), trade complementarity index (TCI), trade intensity index (TII), trade combining density (TCD), and trade specialization index (TSI). Bilateral trade potential is inferred by analyzing the results of the trade indices. The following measurements of potential were taken: the trade potential of 24 agricultural products between China and Kazakhstan was measured by the NRCA, TCI, TCD, and TSI [6,7]; the trade potential of aquatic products between China and ASEAN was analyzed using the TCI and export similarity index (XS) [8]; the trade potential of agricultural products between China and Southeast Asia was analyzed using the trade intensity index (TII) [9]; China’s agricultural trade potential with Central and Eastern Europe was analyzed using constructed complementarity indices [10]; and the agricultural trade potential between China and countries along the 21st Century Maritime Silk Road was analyzed using the trade intensity index (TII) [11].

However, the index method does not take into account in-depth factors such as trade size, production, distribution, and economy when measuring trade potential. Therefore, scholars have begun to use the trade gravity model approach to measure trade potentials. Firstly, scholars have measured the trade potential of agricultural products between China and ASEAN [12], China and Central Asia [13], China and countries along the Silk Road Economic Belt [14], and China and B&R countries [15] using the traditional gravity model and the extended gravity model. The stochastic frontier gravity model is more refined than the traditional gravity model since it represents only the predicted value, not the optimal value; the stochastic frontier gravity model is in the absence of trade resistance, i.e., the optimal value. In recent years, the stochastic frontier gravity model has been the most commonly used method for estimating trade potential, using the level of trade efficiency to measure the size of trade potential. It has been used to measure the following: the potential of agricultural product trade between China and Kazakhstan [16], the potential for agricultural trade between China and RCEP countries [17,18,19], the potential for trade of agricultural products between China and Asian emerging market countries [20], the potential for agricultural trade between China and B&R countries [21,22], the potential for agricultural trade between China and Central Asia [23], and China’s agricultural trade potential with the “Silk Road Economic Belt” countries [24].

The existing literature, which provides ideas for the research in this paper, is still deficient. Firstly, the existing literature utilizes the index method, and most studies infer trade potential through a bilateral trade structure of complementarity and competitiveness, not taking into account the scale of bilateral trade and price factors, especially under the uncertainty of the external international environment. This disrupts the existing terms of trade, and the size of the potential of the trade demand cannot be measured by using the index method.

Alternatively, the stochastic frontier gravity model, a widely utilized method for assessing trade potential, considers factors such as bilateral trade agreements, distance, population, and economic size. It translates these variables into trade efficiency indicators to evaluate the magnitude of the potential trade. However, since the efficiency of international trade is challenging to alter in the short term, the stochastic frontier gravity model is limited to assessing long-term trade potential. Consequently, it cannot effectively measure the short-term demand potential of bilateral agricultural trade, particularly in the context of price fluctuations in the international market.

Therefore, this paper adopts the approach of Xiang (2019) [25] to employ the demand elasticity model for assessing the demand potential of agricultural products between China and B&R countries. Using bilateral relative trade size to represent the structural factors influencing trade volume, along with the magnitude of the import demand elasticity and substitution elasticity to assess the marginal factors related to export competition and price fluctuations, this paper measures trade potential in an innovative manner. Compared with the existing literature, this paper takes into account the interactive competitive substitution relationships between trading countries both within and outside the international trade region. It evaluates and compares the elasticity of substitution, competitiveness, and trade potential of China relative to its major competitors, focusing on price competitiveness rather than market share. This approach enhances our understanding of China’s competitive dynamics with various competitors in its target export countries.

Secondly, regarding the content, Xiang (2019) [25] employed a demand elasticity model to assess China’s export potential to B&R countries. Feng (2021) [26,27] assessed China’s agricultural export potential to the ASEAN region and to several B&R countries by employing a demand elasticity model. However, only select regions within the B&R countries were sampled, and the analysis focused solely on the export potential, neglecting the assessment of the import potential.

Finally, in the measurement procedure for demand potential, Xiang (2019) [25] and Feng (2021) [26,27] do not rank the major trading countries among B&R countries and exclude those with smaller trade volumes because, according to the calculation model of demand elasticity potential, the demand potential is equal to the relative trade size and the elasticity of substitution index multiplied by the elasticity of demand index. However, if the bilateral trade size is very small, short-term market shocks or fluctuations in conditions can prevent a significant increase in demand potential, even if the elasticity of substitution index and the elasticity of demand index are both high. Consequently, the results of the measurement are inevitably inaccurate. Therefore, based on the ranking of agricultural trade between China and B&R countries, this paper assesses the demand for agricultural imports and exports between China and the major B&R countries.

## 3. Evolution of Agricultural Trade Status of B&R Countries and Sample Selection

### 3.1. Evolution of the Ranking of Agricultural Imports of B&R Countries

Screening out the B&R countries with a large share of trade is essential for accurately assessing China’s trade demand potential in B&R countries. Based on the FAO database, this paper analyzes the top 20 imported agricultural products among B&R countries, as presented in Table 1. The import distribution of agricultural products among B&R countries is highly concentrated, primarily involving Russia, India, Vietnam, Poland, Saudi Arabia, Indonesia, Malaysia, Thailand, Turkey, Singapore, the Philippines, Egypt and the Czech Republic. In the past five years, the import value of agricultural products from B&R countries has remained relatively stable. Although it has increased annually, the growth rate has been gradual, rising from USD 334.419 billion in 2017 to USD 424.235 billion in 2021, resulting in an average annual growth rate of 5.37%. From the perspective of the top 20 trade rankings, the share of imports from B&R countries has remained stable at approximately 77% over the past five years.

This paper will focus on the top 20 countries, as they serve as a representative sample for assessing demand potential. The top 10 countries in trade ranking accounted for over 50% of the imports of B&R countries over the past five years. Therefore, selecting these top 10 countries based on their import volume as the focus of cooperation between China and B&R countries is of significant practical importance. At the same time, operationally, the extensive volume and diversity of trade between major agricultural countries and the global market, including China, provide a sufficiently representative sample for calculating elasticity of demand and the elasticity of substitution. Consequently, the results of these calculations are more broadly applicable.

### 3.2. Evolution of the Ranking of Agricultural Exports of B&R Countries

In order to identify potential targets that can meet China’s demand for agricultural imports, we have compiled a ranking of B&R countries that export agricultural products, given in Table 2. This allows us to select the largest agricultural trading countries as key representatives. Agricultural exporting among B&R countries is highly concentrated and stable. The major agricultural exporters in this group include Indonesia, India, Poland, Thailand, Russia, Vietnam, Malaysia, Ukraine, Turkey, Singapore, and Hungary. Over the past five years, the total value of agricultural products exported by B&R countries has increased annually, although it has remained stable overall. Exports grew from USD 334.419 billion in 2017 to USD 454.321 billion in 2021, reflecting an average annual growth rate of 7.17%. In the context of the top 20 exporting countries, these nations accounted for over 88% of the total agricultural exports from B&R countries over the past five years. From the export rankings of the top 10 countries, which account for over 70% of agricultural exports from B&R countries over the past five years, this paper selects these ten countries as representative examples of China’s potential import demand in agricultural trade.

### 3.3. Sample Selection of Agricultural Trade Demand between China and B&R Countries

In order to synthesize the import, export, and total trade indicators, we selected the potential object of the elasticity of demand for agricultural trade between China and B&R countries, as presented in Table 3. According to the analysis of Table 1 and Table 2, the import and export trade of agricultural products of B&R countries has been growing steadily, with a relatively stable ranking. Therefore, the years 2020 and 2021 have been selected as representative samples for this analysis. Since the top 10 importers among B&R countries account for approximately 53% of the total trade share, and the top 10 exporters represent 70% of the overall share, this paper aims to provide a broader perspective on the trade dynamics of B&R countries. To achieve this, it calculates the ranking of the top 20 B&R countries based on total trade, using the importing countries as a benchmark.

According to Table 3, the top 10 B&R countries in 2021 and 2020 remain largely consistent, with eight countries—India, Indonesia, Poland, Russia, Vietnam, Thailand, Malaysia, and Turkey—ranking among the top 10 in terms of imports, exports, and total trade volume. Ukraine ranked 18th in imports, 8th and 7th in exports, and 9th in total trade in both 2021 and 2020. Only Singapore and Saudi Arabia are ranked 10th and 11th in 2021 and 11th and 10th in 2020, respectively. Singapore’s import and export trade rankings are relatively stable, with both import and export rankings at 10th in 2021 and 11th and 13th 2020. In contrast, Saudi Arabia was ranked 5th for imports and 22nd for exports in 2021, while in 2020, it was ranked 5th for imports and 21st for exports. The differences in rankings are significant.

Therefore, from the perspective of import and export equilibrium, Singapore has been selected as one of the top 10 countries for analysis. From the perspective of share, when India, Indonesia, Poland, Russia, Vietnam, Thailand, Malaysia, Turkey, Ukraine, and Singapore are considered as the research subjects, the share of imports in 2021 constituted 50.55% of the total share of the B&R countries, and the top ten export rankings remained unchanged at 72.08%. Therefore, the research sample demonstrates a significant degree of representativeness and feasibility.

## 4. Materials and Methods

### 4.1. Elasticity of Substitution Estimation Methods

In the export market, countries competing with one another have gained market share from their partners. However, relative to the importing country, if a country’s exports are more elastic in substitution compared to its competitors, then the exporting country’s bid will be lower than that of its competitors. As a result, the importing country can acquire the same product at a lower price, leading to a greater share of imports from the exporting country with higher elasticity of substitution. This phenomenon results in a transfer of market share. In this paper, we reference Shiells et al. (1986) [28] and employ the following model to estimate the elasticity of substitution between a country’s exports and those of its competitors in a specific regional market.

(1) $$\ln\left(\frac{E_{it}}{E_{jt}}\right)=C+\sigma *\ln\left(\frac{P_{it}}{P_{jt}}\right)+g*TIME+\varepsilon_{it}$$

In this context, i represents the exporting country, j denotes other exporting competitors in a given region, and ln indicates the natural logarithm. $E_{it}$ is the quantity of agricultural exports from country i to a given region in period t. $E_{jt}$ is the quantity of agricultural products exported to the region by other competing countries in period t, while $\frac{E_{it}}{E_{jt}}$ represents the ratio of market shares of agricultural products exported by country i to country j in period t. $P_{it}$ is the average price of agricultural products exported from the region by country i in period t. $P_{jt}$ is the average price of agricultural exports from other competing countries to the region in period t. $\frac{P_{it}}{P_{jt}}$ represents the intraregional export price ratio of country i to country j in period t. σ is the size of country i’s elasticity of substitution for agricultural exports from other competing countries in the region. TIME is a time-trend variable to represent non-price factors such as excess productivity of agricultural products. $\varepsilon_{it}$ is the randomized perturbation term. It should be noted that the specific region could be the world, the Belt and Road region or a particular country.

First, the elasticity of substitution for China and other exporting countries regarding their competitors’ agricultural products is calculated. Then, the competitor’s market share of agricultural products in a region is multiplied by a weight to calculate a weighted average elasticity of substitution. Finally, the weighted average elasticity of substitution can be utilized to assess the average proportion of trade that can be redirected from competitors when the price of a country’s agricultural exports is comparatively lower in a specific region.

### 4.2. Methodology for Estimating the Elasticity of Demand for Imports

In the international market, the competitive landscape for exporting a country’s agricultural products is influenced not only by competitors within the same sector, but also by the rivalry between the agricultural products of the exporting nation and the domestic agricultural products of the importing nation. Price competition for a country’s agricultural exports can result in increased competition and substitution for domestic demand for agricultural products in importing nations. For the relationship between the two in the context of the game, we adopt the approach of Soderbery (2015) [29] to quantify the import demand elasticity measure. We assume the existence of consumers with nested constant elasticity of substitution (CES) preference structures. For a given level of diversity in the same product, utility can be expressed as:

(2) $$X_{gt}={\left(\sum_{v\in V}\;b_{gvt}^{\frac{1}{\sigma_{g}}}\right)}^{\frac{\sigma_{g}}{\sigma_{g}-1}}$$

This can lead to a demand for diversity in a particular product:

(3) $$s_{gvt}\equiv \frac{p_{gvt}x_{gvt}}{\sum_{v\in I_{gt}}\;p_{gvt}x_{gvt}}={\left(\frac{p_{gvt}}{\emptyset_{gt}(b_{gt})}\right)}^{1-\sigma_{g}}b_{gvt}$$

The set of diversities v of product g in period t can be denoted as $I_{gt}\in \{1\;,\ldots ,\;N\}$, and $x_{gvt}$ is the total amount of diversity v that is consumed in period t product g additively. $\sigma_{g}>1$ is the product g-specific constant elasticity of substitution. $s_{gvt}$ is market share, which depends on its price $p_{gvt}$. A specific stochastic taste parameter $b_{gvt}$ and the minimum production cost of product g subject to the entire vector of taste parameters $\emptyset_{gt}(b_{gt})$. Assuming a monopolistically competitive export market structure, consumers will encounter an upward-sloping supply curve.

(4) $$p_{gvt}=(\frac{\sigma_{g}}{\sigma_{g}-1})exp(\eta_{gvt}){\left(x_{gvt}\right)}^{\omega_{g}}$$

In this context, $\eta_{gvt}$ represents a stochastic technical factor that is independent of the taste parameter $b_{gvt}$.$\omega_{g}>0$ represents the inverse of the elasticity of supply of product g. In order to eliminate time-dependent unobservables, it is first necessary to difference prices and market shares. After applying primary differencing, unobservable factors associated with the product may still persist. These factors can be addressed through second differencing, utilizing the prices and markets of the reference country (the first difference utilizes ∆, and the second difference is denoted by the superscript k), yielding the following structural equations:

(5) $$\Delta^{k}lns_{gvt}\equiv ∆lns_{gvt}-∆lns_{gvkt}=-\left(\sigma_{g}-1\right)\Delta^{k}\ln\left(p_{gvt}\right)+\varepsilon_{gvt}^{k}$$

(6) $$\Delta^{k}lnp_{gvt}\equiv ∆lnp_{gvt}-∆lnp_{gkt}=\left(\frac{\omega_{g}}{1+\omega_{g}}\right)\Delta^{k}\ln\left(s_{gvt}\right)+\delta_{gvt}^{k}$$

where $\varepsilon_{gvt}^{k}=\Delta^{k}ln(b_{gvt})$, $\delta_{gvt}^{k}=\Delta^{k}(\frac{\eta_{gvt}}{1+\omega_{g}})$ are unobservable supply and demand shocks. Definition: $p_{g}\equiv \frac{\omega_{g}\left(\sigma_{g}-1\right)}{1+\omega_{g}\sigma_{g}}\in \left[0,\frac{\sigma_{g}-1}{\sigma_{g}}\right)$. This assumes that the error term $\varepsilon_{gvt}^{k}$ of the difference-processed demand equation and the error term $\delta_{gvt}^{k}$ of the supply equation are independent of each other in time and product space. Simplifying by multiplying (5) and (6) yields the following easily handled equation:

(7) $$Y_{gvt}=\theta_{1}X_{1gvt}+\theta_{2}X_{2gvt}+u_{gvt}$$

where $Y_{gvt}\equiv {\left({∆}^{k}lnp_{gvt}\right)}^{2}$, $X_{tgvt}\equiv {\left({∆}^{k}lns_{gvt}\right)}^{2}$, $X_{2gvt}\equiv ({∆}^{k}lns_{gvt})({∆}^{k}lnp_{gvt})$, $u_{gvt}\equiv \frac{\varepsilon_{gvt}^{k}\delta_{gvt}^{k}}{1-p}$. Because $\theta_{1}\equiv \frac{p}{{\left(\sigma_{g}-1\right)}^{2}(1-p)}$, $\theta_{2}\equiv \frac{2p-1}{\left(\sigma_{g}-1)(1-p\right)}$. After estimating $\theta_{1}$ and $\theta_{2}$ through Equation (7), the estimates of $\sigma_{g}$ and p, and hence $\omega_{g}$, can be found accordingly. Estimates of the elasticity of demand for imports 1 − $\sigma_{g}$ and the elasticity of supply of imports 1ωg are then obtained. This is undertaken by defining the customs HS 6-digit code for agricultural products as product g. The same agricultural product but from different countries is defined as v. Market share $s_{gvt}$ is obtained by dividing the quantity of agricultural product g imported by an importing country from an exporting country v by the total quantity of agricultural product g imported by that importing country. According to the methodology of Feenstra (1994) [30] and Soderbery (2015) [29], this paper identifies the countries whose imported agricultural products represent the largest market share. These countries serve as reference points for the imported agricultural products of the importing nations.

### 4.3. Methodology for Estimating Trade Demand Potential

In the international market, price competition is one of the key strategies for gaining export market share. If an exporting country reduces the price of its agricultural exports in a specific region compared to its competitors, two effects arise. First, importing countries that engage with the exporting country will experience an increase in their demand for agricultural imports due to the lower prices offered by the exporting country. This phenomenon is referred to as the “demand creation effect”. The magnitude of the increase can be quantitatively estimated using the elasticity of demand for imports. Second, when an exporting country reduces the export prices of agricultural products, it can lead to a decrease in imports from other competitors in the importing country. This shift in demand transfers market share to the exporting country, resulting in lower prices for its products. The extent of the impact of this phenomenon can be estimated using the elasticity of substitution. Competition in the international market hinges on the pursuit of trade share. If a country’s trade share is limited, even a significant demand creation effect and demand transfer effect may not suffice to meet the needs of importing countries. Therefore, it is essential to consider three factors: the import demand elasticity, the elasticity of substitution, and the relative size of imports. The product of these three factors serves as a metric for assessing export competition in the context of trade potential measurement index. In this paper, we adopt the methodology proposed by Xiang (2019) [25] to quantify trade demand potential using the product of these three factors, which is formulated as follows:

(8) $$TP=E_{s}\times E_{d}\times IS$$

where TP is the demand potential for export trade, $E_{s}$ is the elasticity of substitution for exports, $E_{d}$ is the elasticity of demand for imports, and IS is the relative size of imports.

### 4.4. Data Sources and Data Characterization

In this paper, the agricultural trade volumes are sourced from the UN Comtrade database, while the data on agricultural trade prices are obtained from the CEPII-BACI database. In order to analyze the evolution of agricultural trade elasticity following China’s accession to the World Trade Organization, this paper examines a time frame from 2001 to 2021. Considering B&R countries collectively, which encompass over 60 countries, the scale of their agricultural trade is nearly four times that of China. In the international market, China competes with the world’s leading agricultural exporters for a share of imports from B&R countries. According to the FAO database, in addition to China, the top five agricultural exporters in 2021 were the United States, the Netherlands, Brazil, Germany, and France. Therefore, this paper selects the United States, the Netherlands, Brazil, Germany, and France as the primary competitors for China’s exports to B&R countries.

We compared China’s export price competition with that of the United States, the Netherlands, Brazil, Germany and France (the Big Five countries). At the same time, 10 B&R countries—namely India, Indonesia, Poland, Russia, Vietnam, Thailand, Malaysia, Turkey, Ukraine, and Singapore—were selected as representatives of the B&R countries to compare price competition between B&R countries and the export markets of the United States, the Netherlands, Brazil, Germany, and France. This paper selects the top 40 countries in the world and B&R countries scope of agricultural import and export trade as the sample size, representing 81.98% and 87.29% of the total amount of import and export in the world and 95.63% and 98.72% of the total amount of import and export in B&R countries. Therefore, the research sample is considered representative, and the results are presented in Table 4.

According to Table 4, in the world and B&R countries, China has the lowest relative price of agricultural exports compared to the United States, the Netherlands, Brazil, Germany, and France. This is a price advantage that makes it more challenging for China to increase its market share through a price reduction strategy. In the world, among B&R countries, Thailand exhibits the highest relative price, while Ukraine has the lowest. However, when compared to competitors such as the United States, the Netherlands, Brazil, Germany, and France, the relative prices of exports from B&R countries are generally lower than those of the United States, Germany, and France. Notably, only Thailand’s relative price exceeds that of the Netherlands, and the relative prices of India and Indonesia are higher than Brazil’s. Overall, this indicates that the prices of agricultural products exported by B&R countries are relatively low. However, for a country’s exports, the ability to leverage lower export prices in the market compared to other competitors, as well as the capacity to redirect demand for imports away from competitors in order to capture a larger share of exports, is the ultimate objective. Consequently, this paper will employ the elasticity of substitution to quantify the extent of trade diversion capacity for China, the Big Five countries, and the B&R countries.

## 5. Results of Measuring the Demand Potential of Agricultural Trade between China and B&R Countries

### 5.1. Results of Elasticity of Substitution Measures

Using Equation (1), the elasticity of substitution is calculated by regressing the top five competitors of China’s exports in the world and the B&R countries, specifically, the United States, the Netherlands, Brazil, Germany, and France. The results are presented in Table 5. Combined with Table 5, it can be confirmed that China has the lowest relative prices, and all elasticities of substitution are less than 1. In the world market, the elasticity of substitution for China’s agricultural exports in relation to most competing countries is less than 1. This indicates that China’s strategy of reducing the prices of its agricultural exports in the world market in order to capture a shifted market share of import demand has not been particularly effective. The reason for this is that, in the world, compared to developed countries, China’s agricultural export prices are already relatively low. Consequently, the ability to further reduce prices and stimulate a shift in demand for agricultural products from importing countries is limited. At the same time, the elasticity of substitution for China’s agricultural exports compared to most of its major competitors in the markets of B&R countries is found to be less than 1 percent. China’s capacity to capture a share of imports from the top five B&R countries by reducing the export prices of its agricultural products is limited in the markets encompassed by these B&R countries. The specific reason is that China’s elasticity of substitution is less than 1 in the world. This is because China’s export prices are already low, and the marginal effect of increasing market share through lower prices is minimal.

According to the same methodology, the scope of this study is confined to B&R countries. The average elasticity of substitution for the top ten agricultural exports of China and the five major competitors within the B&R countries is calculated as a measure of the competitive substitution ability of the B&R countries, as summarized in Table 6. A vertical comparison of China’s exports to B&R countries, the top ten B&R countries as a whole, reveals a weak demand transfer effect. China’s weighted average elasticity of substitution for nearly half of the B&R countries is less than 1, with the top five being Vietnam, Indonesia, Russia, Turkey, and Poland. When comparing China with the five major competing countries, it is observed that the weighted average elasticity of substitution for China, Brazil, and the Netherlands is low, whereas the elasticity for the United States, Germany, and France is high. According to Table 4, the export prices of China, Brazil, and the Netherlands are relatively low. As a result, the share of demand that can be shifted by lowering prices is limited, leading to a relatively small index.

Following the same methodology, this study is limited to China. The average elasticity of substitution for B&R countries and the five largest competitors in China is calculated separately, as shown in Table 7. The weighted average elasticity of substitution of most of the major B&R countries exporting to China is less than 1. This includes Vietnam, Ukraine, Indonesia, Poland, Thailand, and Russia. These countries do not export agricultural products at high prices in the world market, and their capacity to reduce import prices of agricultural products to compete for competitors’ market share is inadequate. As a result, the demand-shifting effect is weak. The countries with a weighted average elasticity of substitution greater than 1 are India and Malaysia. The weighted average elasticity of substitution among the other five largest competitors exceeds 1, suggesting that price reductions could significantly shift a larger portion of China’s trade share within the market. The conclusion drawn from the data presented in Table 4 is that the U.S., the Netherlands, Brazil, Germany, and France possess higher relative prices in the world market. Consequently, these countries can capture a larger market share from their competitors when the Big Five countries reduce their export prices.

### 5.2. Results of Estimation of Import Demand Elasticity

This paper employs the elasticity of demand for agricultural imports as an indicator to measure the changes in the volume of agricultural imports from importing countries due to fluctuations in import prices in China and major B&R countries. The greater the elasticity of demand for agricultural imports, the more responsive an importing country’s imports are to price reductions in the exporting country. Consequently, the larger the price reductions in the exporting country’s agricultural products, the more it can increase the volume of exports to the importing country. In this paper, based on Equation (7), the elasticity of demand for agricultural imports from major B&R countries to China, as well as the elasticity of demand for agricultural exports from China to B&R countries, are calculated.

Based on Table 8, B&R countries of China’s import demand elasticity, the weighted average ranking of the top five were Ukraine, Turkey, Poland, Russia, and India. This indicates that these five countries reduce the price of agricultural products by importing more agricultural products from China, but the elasticity of demand of Singapore and Vietnam is less than 1, indicating that in these two countries, it is difficult to reduce the price of agricultural products by increase the volume of exports from China. In the measurement results, B&R countries that have a large trade volume of agricultural products have a relatively large number of species. Malaysia boasts the highest number, with 319 species, while Thailand has the lowest, with 86 species. Therefore, the findings of this study are representative to a certain extent. Among the values analyzed, the minimum demand elasticity is −196.48, while the maximum is 380.88, indicating a significant range of change. However, the change in the mean value is relatively small, with the maximum and minimum mean values being 10.53 and 0.94, respectively. These figures suggest the data are stable and credible.

In the context of the elasticity of demand for China’s imports to major B&R countries, the top five in terms of weighted averages are Poland, Russia, Indonesia, Turkey, and India (Table 9). This suggests that China’s lowering of agricultural commodity prices can result in gaining more export shares to these five countries. However, the elasticity of demand of Vietnam and Thailand is less than 1, indicating that these two countries are relatively insensitive to China’s agricultural imports even if China lowers its export prices. Even if China reduces export prices, the two countries remain relatively insensitive to China’s agricultural imports. According to the measurement results, among the main B&R country exports, the number of agricultural trade categories in Thailand is the largest, at 217, while that in Ukraine is the smallest, at 79. These findings suggest that the results of the study are somewhat representative. The minimum value of the elasticity of demand is −361.23, while the maximum value is 266.58, indicating a significant range of change. In contrast, the mean value exhibits a smaller variation, with maximum and minimum values of 9.05 and 0.15, respectively. These values are both more stable and credible.

### 5.3. Results of Trade Demand Potential Measurements

According to Equation (8), China’s agricultural export potential to major B&R countries is assessed, and the results are presented in Table 10. China’s export potential to the main B&R countries is ranked as follows: Indonesia, Thailand, Russia, Poland, Turkey, Vietnam, Malaysia, Ukraine, India, and Singapore. These mainly ranked ahead of the countries importing China’s agricultural products on a relatively large scale, which aligns with the market situation. A larger import scale enables these countries to compete for a greater share of exports. The relative trade size represents the comparative value of imports of major B&R countries of China’s agricultural products. Being the largest importer among the B&R countries, Russia’s imports of China’s agricultural products are at base 1. The relative sizes of the top ten B&R countries, in order, are as follows: Thailand, Indonesia, Vietnam, Malaysia, and Russia. In contrast, countries with smaller size and lower demand potential include Singapore, India, and Ukraine.

Based on Equation (8), the agricultural export potential of major B&R countries to China was assessed, resulting in Table 11. The main B&R countries of China’s export potential rankings are Vietnam, India, Ukraine, Russia, Malaysia, Thailand, Indonesia, Poland, Singapore, and Turkey. In this context, countries with more elastic demand play a significant role, and their relative size also contributes to the rankings. Relative trade size is a comparative measure of the export volume of agricultural products from major B&R countries to China. Since Indonesia is the largest exporter among B&R countries, the export volume of agricultural products from Indonesia to China serves as the base 1. Only Thailand surpasses Indonesia as the first-generation importer of goods from China, while the relative size of other countries exporting to China remains small, leading to a reduced potential for final export demand.

## 6. Discussion

Based on a comparison of existing research results utilizing the trade gravity model, Li Jinkai (2020) [31] concludes that China’s agricultural exports to the “Belt and Road” countries are ranked in terms of trade potential as follows: India, Vietnam, Philippines, Indonesia, Russia, Thailand, Pakistan, Bangladesh, Iran, and Myanmar. Liu Yujun (2021) [32] identifies that China imports B&R countries, including Laos, Belarus, Latvia, Ukraine, Thailand, Singapore, Bosnia and Herzegovina, Turkmenistan, Malaysia, and Estonia. Additionally, China exports to B&R countries: Singapore, Jordan, Malaysia, Russia, Lebanon, Georgia, Vietnam, Thailand, Kyrgyzstan, and Belarus. These findings are largely consistent with the results presented in this paper.

However, Wenyan Song’s (2021) [33] trade gravity model assesses the trade potential of agricultural products between China and the B&R countries. The model identifies the top ten countries with the highest trade potential as follows: Kuwait, Bosnia and Herzegovina, Oman, Moldova, Albania, Qatar, Tajikistan, Slovakia, Armenia, and Azerbaijan. The bottom ten countries in terms of trade potential are New Zealand, Thailand, Malaysia, Vietnam, Singapore, Indonesia, Russia, South Korea, Ukraine, and the Philippines. This ranking is essentially the opposite of the findings presented in this paper.

According to a comparison of scholars utilizing the elasticity of demand approach to trade, the research conducted by Feng Xiaoling [27] identifies countries such as Vietnam, Thailand, Singapore, Russia, and Indonesia and other countries as China’s exports of agricultural products among B&R countries with greater demand potential. These findings are consistent with this paper, indicating that the conclusions of this paper have a certain degree of scientific validity. However, there is a distinction to be made regarding the agricultural trade of the Philippines, Nigeria, and China. Due to their relatively small size, elastic demand cannot be effectively utilized to assess the potential of the bilateral trade. Consequently, this paper does not include these countries in the sample. In contrast, Feng Xiaoling [27] incorporates the Philippines and Nigeria into her research sample, identifying the Philippines as the country with the largest trade potential. Additionally, while Feng only evaluates the agricultural trade demand potential for China’s exports to B&R countries, she does not assess the import demand potential. This paper addresses this gap.

This paper also has limitations, because it only analyzes data from 2021, and does not intercept more time points for horizontal comparison and evolution. Therefore, in the future, it will be possible to quantify the changes in the trade potential of agricultural products between China and the B&R countries since the Belt and Road Initiative was introduced in 2013. Based on this analysis, we can verify and predict the objectives of future bilateral cooperation. On this basis, it is possible to assess and forecast the objectives of future bilateral cooperation. At the same time, it is possible to further categorize and refine the agricultural products under investigation and assess the demand potential for various specific agricultural products between China and the B&R countries. Since various crops exhibit distinct growth characteristics, according to the trade potential of different specific agricultural products, it is required to find a more tailored foundation for formulating policies that promote cooperation between China and the B&R countries.

At a time when the Russian–Ukrainian conflict persists, and global climate change contributes to uncertainty in the world’s food production and distribution, measuring the potential of China’s trade in agricultural products with major B&R countries will help to ensure food security for the vast majority of developing nations. Simultaneously, it will enhance agricultural production and create employment opportunities for farming households in these developing countries.

Among the top ten countries with significant trade potential, ASEAN nations account for 4–5. Notably, on 23 May 2023, during the Sixth Western China International Investment and Trade Fair, the “China-ASEAN Investment and Trade Legal Policy Guidelines” was officially released, marking a more mature cooperation between China and ASEAN countries. Building on this foundation, China should prioritize strengthening agricultural ties with Vietnam, Malaysia, Thailand, Singapore and other ASEAN nations. It should provide essential support in areas such as agricultural investment, trade settlement, the procurement of agricultural machinery, and other policies aimed at promoting China’s agricultural production and trade in agricultural products with key ASEAN nations.

Among the top ten potential trade partners, Russia is a significant agricultural partner for China, combined with China’s domestic Free Trade Agreement (FTA) strategy, especially the Heilongjiang FTA. Due to the geographic advantages of Heilongjiang’s proximity to the Russian Federation, particularly its abundant natural arable land, there exists a significant opportunity for agricultural collaboration. While Russia has vast areas of fertile land, it also has considerable barren regions. Heilongjiang, recognized as China’s leading agricultural province, possesses extensive experience in large-scale agricultural operations and the use of advanced machinery and equipment. Therefore, it is essential to integrate China’s agricultural expertise and equipment with Russia’s resources to enhance agricultural productivity. This collaboration can be further promoted through the Heilongjiang FTA, which aims to bolster China–Russia agricultural trade.

## 7. Conclusions

This chapter analyzes trade demand potential by breaking it down to three influencing factors: relative size, import demand elasticity, and export substitution elasticity. This analysis quantifies the impact of these factors on the major B&R countries and ultimately assesses the trade demand potential of China and the top ten B&R countries.

In terms of the scale of trade demand, Russia, India, Vietnam, Poland, Saudi Arabia, Indonesia, Malaysia, Thailand, Turkey, Singapore, etc., are the major importers among B&R countries, accounting for more than 54% of the total imports of B&R countries in 2021. Similarly, Indonesia, India, Poland, Thailand, Russia, Vietnam, Malaysia, Ukraine, Turkey, and Singapore are the leading exporters among B&R countries, representing over 72% of the total exports of B&R countries in 2021.

In the world and in B&R countries, China’s agricultural exports exhibit the lowest relative prices when compared to those of the United States, the Netherlands, Brazil, Germany, and France, indicating a price advantage for China. When the main B&R countries are compared to the United States, Germany, and France, the prices of the latter are lower, while only Thailand’s relative price is higher than that in the Netherlands, Brazil, India, Indonesia, and Brazil among the B&R countries. Overall, the relative prices of agricultural exports from B&R countries tend to be low.

China’s exports to major B&R countries exhibit a weak the demand transfer effect. The weighted average elasticity of substitution for nearly half of B&R countries is less than 1. The top five countries in this regard are Vietnam, Indonesia, Russia, Turkey, and Poland. The weighted average elasticity of substitution of most for B&R countries exporting to China is less than 1. Specifically, this includes Vietnam, Ukraine, Indonesia, Poland, Thailand, and Russia. As a result, the demand transfer effect is weak, and they have the ability to reduce the import prices of agricultural products to compete for market share against their competitors. In contrast, countries with a weighted average elasticity of substitution greater than 1 include India and Malaysia.

In terms of the elasticity of demand for China’s imports from B&R countries, the top five, based on weighted averages, are Ukraine, Turkey, Poland, Russia, and India. In the elasticity of demand for China’s imports to major B&R countries, the top five, also based on weighted averages, are Poland, Russia, Indonesia, Turkey, and India.

China’s agricultural export potential to major B&R countries is ranked as follows: Indonesia, Thailand, Russia, Poland, Turkey, Vietnam, Malaysia, Ukraine, India, and Singapore. Conversely, the export potential of major B&R countries to China is ranked as follows: Vietnam, India, Ukraine, Russia, Malaysia, Thailand, Indonesia, Poland, Singapore, and Turkey.

## Figures and Tables

**Table 1:** Evolution of the ranking of agricultural imports of the top 20 B&R countries, 2017–2021 Unit: billion USD.

Year Country	2021		2020		2019		2018		2017	
	Imports	Ranking	Imports	Ranking	Imports	Ranking	Imports	Ranking	Imports	Ranking
Russia	315	1	273	1	282	1	280	1	277	1
India	289	2	204	3	191	3	195	3	251	2
Vietnam	282	3	191	4	182	4	183	4	166	5
Poland	269	4	231	2	209	2	213	2	181	4
Saudi Arabia	233	5	188	5	180	5	179	6	195	3
Indonesia	215	6	170	6	172	6	183	5	163	6
Malaysia	191	7	156	7	146	8	149	7	142	7
Thailand	183	8	150	8	132	10	141	9	133	9
Turkey	167	9	140	9	134	9	120	12	125	10
Singapore	1578	10	122	11	121	12	122	10	118	11
Philippines	151	11	119	12	128	11	119	11	103	12
Egypt	147	12	135	10	159	7	148	8	134	8
Czech Republic	125	13	99	13	98	13	96	13	90	13
Romania	120	14	94	14	86	14	82	14	76	14
Pakistan	95	15	69	15	54	17	60	16	68	15
Israel	88	16	64	17	61	16	58	17	55	17
Hungary	84	17	64	16	62	15	60	15	56	16
Ukraine	76	18	60	18	53	18	46	20	39	20
Slovakia	64	19	51	19	50	20	50	19	45	19
Kuwait	54	20	54	20	53	19	52	18	48	18
Total Belt and Road countries	4242	-	3442	-	3302	-	3289	-	3169	-
Total of top 20 countries	3305	-	2633	-	2554	-	2536	-	2465	-
Total of top 10 countries	2302	-	1825	-	1750	-	1764	-	1751	-

Data source: Authors’ compilation based on FAO database.

**Table 2:** Evolution of the ranking of agricultural exports of the top 20 B&R countries, 2017–2021 Unit: billion USD.

Year	2021		2020		2019		2018		2017	
Country	Exports	Ranking	Exports	Ranking	Exports	Ranking	Exports	Ranking	Exports	Ranking
Indonesia	491	1	352	2	309	4	335	2	357	1
India	450	2	354	1	340	1	345	1	349	2
Poland	402	3	351	3	321	3	324	4	276	4
Thailand	382	4	315	4	326	2	331	3	310	3
Russia	327	5	274	5	241	6	242	6	201	7
Vietnam	287	6	250	6	253	5	262	5	264	5
Malaysia	275	7	204	8	190	8	191	7	203	6
Ukraine	267	8	220	7	219	7	184	8	176	9
Turkey	250	9	198	9	189	9	184	9	179	8
Singapore	145	10	68	13	84	11	85	11	81	11
Hungary	126	11	97	10	93	10	91	10	90	10
Romania	113	12	73	12	74	13	70	13	66	13
Czech Republic	105	13	81	11	75	12	74	12	74	12
Bulgaria	71	14	53	16	52	16	50	17	46	17
Lithuania	71	15	66	14	58	15	55	15	51	15
Philippines	66	16	61	15	65	14	59	14	64	14
Egypt	61	17	48	18	51	18	48	18	47	16
Pakistan	54	18	46	19	52	17	54	16	44	18
Serbia	50	19	39	20	34	20	32	20	30	20
Myanmar	49	20	48	17	44	19	46	19	44	19
Total Belt and Road countries	4543	-	3626	-	3485	-	3467	-	3344	-
Total of top 20 countries	4041	-	3198	-	3070	-	3059	-	2953	-
Total of top 10 countries	3275	-	2587	-	2472	-	2482	-	2396	-

Data source: Authors’ compilation based on FAO database.

**Table 3:** Evolution of agricultural trade ranking of the top 20 B&R countries in 2020–2021 Unit: billion USD.

Year	2021							2020							
Country	Imports	Ranking	Exports	Ranking	Country	Total Trade	Ranking	Country	Ranking	Ranking	Exports	Ranking	Country	Total Trade	Ranking
Russia	315	1	327	5	India	739	1	Russia	272	1	274	5	547	Poland	1
India	289	2	449	2	Indonesia	706	2	India	203	3	353	1	557	India	2
Vietnam	282	3	286	6	Poland	670	3	Vietnam	190	4	250	6	440	Russia	3
Poland	268	4	401	3	Russia	642	4	Poland	231	2	350	3	582	Indonesia	4
Saudi Arabia	233	5	40	22	Vietnam	568	5	Saudi Arabia	188	5	35	21	223	Thailand	5
Indonesia	215	6	490	1	Thailand	564	6	Indonesia	170	6	351	2	522	Vietnam	6
Malaysia	190	7	275	7	Malaysia	466	7	Malaysia	155	7	204	8	360	Malaysia	7
Thailand	182	8	381	4	Turkey	416	8	Thailand	149	8	315	4	465	Turkey	8
Turkey	167	9	250	9	Ukraine	343	9	Turkey	140	9	198	9	339	Ukraine	9
Singapore	158	10	145	10	Singapore	302	10	Singapore	122	11	68	13	190	Saudi Arabia	10
Philippines	151	11	66	16	Saudi Arabia	274	11	Philippines	119	12	61	15	180	Singapore	11
Egypt	147	12	61	17	Romania	233	12	Egypt	135	10	48	18	183	Egypt	12
Czech Republic	125	13	105	13	Czech Republic	229	13	Czech Republic	99	13	81	11	180	Czech Republic	13
Romania	120	14	113	12	Philippines	217	14	Romania	94	14	73	12	166	Philippines	14
Pakistan	95	15	54	18	Hungary	210	15	Pakistan	69	15	46	19	115	Romania	15
Israel	88	16	23	29	Egypt	208	16	Israel	64	17	20	29	83	Hungary	16
Hungary	84	17	126	11	Pakistan	149	17	Hungary	64	16	97	10	160	Pakistan	17
Ukraine	76	18	267	8	Israel	111	18	Ukraine	60	18	219	7	280	Slovakia	18
Slovakia	64	19	42	21	Slovakia	106	19	Slovakia	50	19	34	23	85	Israel	19
Kuwait	54	20	3	46	Kuwait	57	20	Kuwait	54	20	4	45	58	Kuwait	20

Data source: Authors’ compilation based on FAO database.

**Table 4:** Relative prices between China and major trading countries in the world and B&R countries.

Relative Prices of China’s Exports to Its Main Competitors						Relative Prices of Exports from B&R Countries to Major Competitors					
Country	Observed	Mean	S.D.	Max.	Min.	Country	Observed	Mean	S.D.	Max.	Min.
p-world US	40	2.36	1.33	7.90	0.23	p-world IN	40	1.27	0.71	4.18	0.12
p-world NL	40	1.30	0.74	4.36	0.13	p-world ID	40	1.29	0.72	4.27	0.12
p-world BR	40	1.26	0.71	4.21	0.12	p-world PL	40	0.59	0.33	1.94	0.06
p-world DE	40	1.64	0.92	5.47	0.16	p-world RU	40	0.80	0.45	2.64	0.08
p-world CN	40	0.89	0.50	2.97	0.09	p-world VN	40	0.89	0.49	2.93	0.09
p-world FR	40	1.62	0.89	5.36	0.16	p-world TH	40	1.42	0.78	4.70	0.14
p-BR US	40	3.48	2.48	9.41	0.68	p-world MY	40	0.88	0.48	2.90	0.08
p-BR NL	40	1.92	1.57	5.20	0.38	p-world TU	40	0.50	0.28	1.64	0.05
p-BR BR	40	1.86	1.29	5.02	0.36	p-world UA	40	0.30	0.17	1.00	0.03
p-BR DE	40	2.41	1.53	6.52	0.47	p-world SG	40	1.05	0.59	3.47	0.10
p-BR CN	40	1.31	1.34	3.54	0.26	-	-	-	-	-	-
p-BR FR	40	2.38	1.45	7.32	0.43	-	-	-	-	-	-

Note: BR stands for B&R countries region, and the average price is 1. US, NL, BR, DE, CN, FR represent the United States, the Netherlands, Brazil, Germany, China and France, respectively. IN, ID, PL, RU, VN, TH, MY, TU, UA, SG represent India, Indonesia, Poland, Russia, Vietnam, Thailand, Malaysia, Turkey, Ukraine and Singapore, respectively. p-world US stands for the U.S. agricultural price ratio in the world, and the rest of the variables follow suit.

**Table 5:** Coefficient of elasticity of substitution of China’s agricultural exports to its main competitors in the world and in the Belt and Road context Es.

Coefficient of Elasticity	China-USA		China-Netherlands		China-Pakistan		China-Germany		China-France	
	World	BR	World	BR	World	BR	World	BR	World	BR
Ln(pi/pj)	−0.891 **	−0.874 ***	−0.933 **	−0.945 ***	−0.972	−0.981 *	−0.902 ***	−0.931 **	−0.924 **	−0.965 **
	(−1.23)	(−0.11)	(1.30)	(1.39)	(0.33)	(1.91)	(4.39)	(0.77)	(2.45)	(1.38)
Obs	21	21	21	21	21	21	21	21	21	21
R^2^	0.919	0.875	0.885	0.832	0.610	0.841	0.908	0.759	0.961	0.940

Note: Values in parentheses are standard deviations, and ***, **, and * represent 1%, 5%, and 10% significance levels, respectively.

**Table 6:** Weighted average elasticity of substitution of exporters against competitors in B&R countries.

Country	China	United States	The Netherlands	Brazil	Germany	France
Vietnam	1.352	1.406	1.636	1.148	1.451	1.917
Indonesia	1.295	1.534	1.414	1.133	1.341	1.343
Russia	1.144	1.327	0.917	1.148	1.274	0.988
Turkey	1.085	1.169	0.371	1.004	1.254	1.66
Poland	0.973	1.147	1.016	1.026	1.211	1.212
Thailand	0.962	1.206	1.169	0.953	1.253	1.036
India	0.842	1.331	1.004	1.047	1.263	1.66
Ukraine	0.826	1.13	0.379	1.422	1.13	1.086
Malaysia	0.822	0.941	0.814	0.932	1.129	1.019
Singapore	0.739	1.103	1.016	9.861	0.989	1.104

Note: Countries in the first row represent exporting countries, and countries in the first column represent importing countries.

**Table 7:** Weighted average elasticity of substitution Es for each exporter against competitors in China.

Exports of the world’s leading competitor countries	United States	The Netherlands	Brazil	Germany	France					
Elasticity of substitution	1.363	1.222	1.086	1.682	1.504					
Exports from B&R countries	India	Indonesia	Russia	Poland	Vietnam	Thailand	Malaysia	Turkey	Ukraine	Singapore
Elasticity of substitution	1.032	0.843	0.951	0.894	0.712	0.937	1.402	0.866	0.825	0.981

Source of data: compiled by the authors.

**Table 8:** Estimated elasticity of demand for China’s agricultural imports in major B&R countries Ed.

Country	Types of Agricultural	Minimum	Maximum	Mean	Median	Weighted Average
Ukraine	116	−51.15	146.81	10.53	0.09	11.87
Turkey	157	−40.32	215.64	4.35	2.49	5.86
Poland	93	−89.98	42.62	2.62	0.07	5.03
Russia	257	−107.86	151.6	1.61	2.21	4.11
Indonesia	235	−192.81	73.78	6.42	0.17	3.8
India	147	−48.88	72.19	3.59	2.95	3.33
Thailand	86	−85.43	75.42	0.24	1.38	2.94
Malaysia	319	−99.87	380.88	8.95	1.42	1.27
Vietnam	172	−196.48	241.05	2.3	0.41	0.94
Singapore	236	−90.47	116.78	3.01	1.1	0.85

Source of data: compiled by the authors.

**Table 9:** Estimated elasticity of demand for agricultural imports from China to major B&R countries Ed.

Country	Types of Agricultural	Minimum	Maximum	Mean	Median	Weighted Average
Poland	126	−147.21	74.53	5.65	1.42	9.71
Russia	157	−87.52	31.13	1.04	1.33	8.65
Indonesia	168	−89.94	74.79	1.29	1.66	4.83
Ukraine	79	−77.54	164.54	9.49	1.3	4.21
Turkey	107	−68.08	53.2	0.15	1.68	3.15
India	152	−361.23	266.58	3.39	1.85	2.37
Malaysia	173	−311.26	71.65	9.05	1.64	1.07
Singapore	82	−50.85	196.1	4.04	0.49	1.04
Vietnam	189	−93.43	51.17	1.72	0.9	0.51
Thailand	217	−32.25	138.87	3.51	1.2	0.48

Source of data: compiled by the authors.

**Table 10:** Measurement of China’s trade potential in agricultural exports to major B&R countries Initiative.

Country	Relative Size of Imports IS	Elasticity of Demand Ed	Elasticity of Substitution Es	Potential TP	Potential Ranking
Indonesia	1.89	3.8	1.3	9.3	1
Thailand	2.57	2.94	0.96	7.27	2
Russia	1.00	4.11	1.14	4.7	3
Poland	0.34	5.03	0.97	1.66	4
Turkey	0.26	5.86	1.09	1.65	5
Vietnam	1.23	0.94	1.35	1.56	6
Malaysia	1.45	1.27	0.82	1.51	7
Ukraine	0.14	11.87	0.83	1.37	8
India	0.26	3.33	0.84	0.73	9
Singapore	0.66	0.85	0.74	0.41	10

Data source: compiled by the authors.

**Table 11:** Measured demand potential of major B&R countries for China’s agricultural export trade.

Country	Relative Size of Imports IS	Elasticity of Demand Ed	Elasticity of Substitution Es	Potential TP	Potential Ranking
Vietnam	0.68	9.71	0.71	4.7	1
India	0.34	4.83	1.03	1.69	2
Ukraine	0.46	4.21	0.83	1.6	3
Russia	0.37	2.37	0.95	0.83	4
Malaysia	0.35	1.07	1.4	0.53	5
Thailand	1.07	0.51	0.94	0.51	6
Indonesia	1.00	0.48	0.84	0.4	7
Poland	0.02	8.65	0.89	0.15	8
Singapore	0.15	1.04	0.98	0.15	9
Turkey	0.04	3.15	0.87	0.11	10

Data source: compiled by the authors.

## Data Availability

The data presented in this study are available on request from the corresponding author.
